# Correction: Optimized model predictive control for improving dynamic stability and steering accuracy in multi-axle cranes

**DOI:** 10.1371/journal.pone.0339815

**Published:** 2025-12-30

**Authors:** The PLOS One Staff

## Notice of Republication

This article was republished on December 11, 2025, to correct errors in the figures and Funding Information.

The following figure changes were made:

Fig 7 - New file provided

Fig 8 - New file provided

Fig 9 - New file provided

Fig 10 - Replaced with file from Fig 7

Fig 11 - Replaced with file from Fig 8

Fig 12 - Replaced with file from Fig 9

Fig 13 - Replaced with file from Fig 10

Fig 14 - Replaced with file from Fig 11

Fig 15 - Replaced with file from Fig 12

Fig 16 - Replaced with file from Fig 13

Fig 17 - Replaced with file from Fig 14

Fig 18 - Replaced with file from Fig 15

Fig 19 - Replaced with file from Fig 16

Fig 20 - Replaced with file from Fig 17

Fig 21 - Replaced with file from Fig 18

The Funding Information was updated to the following: This research was funded by the Deanship of Research and Graduate Studies at King Khalid University through the Large Research Project grant number RGP2/254/45.

The publisher apologizes for the errors. Please download this article again to view the correct version. The originally published, uncorrected article and the republished, corrected articles are provided here for reference.

## Supporting information

S1 File Originally published, uncorrected article. (PDF)

S2 File Republished, corrected article. (PDF)
