# Supplementary material for: Correction: Optimized model predictive control for improving dynamic stability and steering accuracy in multi-axle cranes
Source: PLoS One. 2025 Dec 30;20(12):e0339815. doi: 10.1371/journal.pone.0339815 (PMC12752948; doi:10.1371/journal.pone.0339815)
Supplement: S2 File — (PDF) [file pone.0339815.s002.pdf]

PLOS One

RESEARCH ARTICLE

Optimized Model Predictive Control for improving dynamic stability and steering accuracy in multi-axle cranes

Abdulhakeem Muhammed Ali[1], Yusuf Abubakar Sha'aban[2,*], Ahmed Tijani Salawudeen[3], Zaharuddeen Haruna[1], Bilyamin Muhammad[4], Muhammed Bashir Mu'azu[1], Abdullah Alharthi[5]

1 Department of Computer Engineering, Ahmadu Bello University, Zaria, Nigeria, **2** Department of Electrical Engineering, University of Hafr Al Batin, Hafr Al Batin, Saudi Arabia, **3** Department of Electrical Engineering, University of Jos, Nigeria, **4** Department of Computer Engineering, Kaduna Polytechnic, Nigeria, **5** Department of Electrical Engineering, King Khalid University, Abhā, Saudi Arabia Arabia

* shaaban@uhb.edu.sa

Check for updates

OPEN ACCESS

Citation: Ali AM, Sha'aban YA, Salawudeen AT, Haruna Z, Muhammad B, Mu'azu MB, et al. (2025) Optimized Model Predictive Control for improving dynamic stability and steering accuracy in multi-axle cranes. PLoS One 20(7): e0324720. https://doi.org/10.1371/journal.pone.0324720

Editor: Lei Zhang, Beijing Institute of Technology, CHINA

Received: September 27, 2024

Accepted: April 30, 2025

Published: July 2, 2025

Copyright: © 2025 Ali et al. This is an open access article distributed under the terms of the Creative Commons Attribution License, which permits unrestricted use, distribution, and reproduction in any medium, provided the original author and source are credited.

Data availability statement: All data are in the manuscript and/or supporting information files.

Funding: This research was funded by the Deanship of Research and Graduate Studies at King Khalid University through the Large Research Project grant number RGP2/254/45.

Abstract

The multi-axle crane, a long vehicle with high inertia, has historically struggled with steering efficiency and path-tracking performance. Various control strategies, including Proportional-Integral-Derivative (PID), Linear Quadratic Regulator (LQR), and Model Predictive Control (MPC), have been employed to address these challenges. However, while improving steering efficiency, these strategies have often led to poor path-tracking performance. This work presents a significant advancement in the form of an optimized MPC for improved steering control of the multi-axle crane. A bicycle model of the multi-axle crane was adopted for the work. MPC was designed, and the smell agent optimization technique (SAO) was employed to optimize the steering input weighting factor, which determines the path-tracking performance. This provided an improved and accurate path-tracking performance for different driving speed conditions. Simulation and performance evaluation of the optimized MPC for the steering system were carried out on a curved road path for three different driving speed scenarios (25, 45, and 65 km/h). The results were compared with existing steering systems that utilized the MPC using steering efficiency, dynamic stability, and path-tracking performance. Results obtained showed improvements of 13.88%, 46.02%, and 18.35% in steering efficiency for the three scenarios over the benchmark scheme. Similarly, improvements of 2.29%, 1.03%, and 4.17%, respectively, were achieved in terms of dynamic stability for the three scenarios. For lateral error, improvements of 26.78%, 26.35%, and 27.52% were achieved, while 27.44%, 29.25%, and 28.93% were achieved for the yaw angle error in the three scenarios, respectively. A 3D simulation model for the multi-axle crane was developed in Any-Logic for visual interpretation and validation of the tracking results. These results

Competing interests: The authors have declared that no competing interests exist.

showed that the developed MPC steering system achieved better steering performance than the existing scheme.

1. INTRODUCTION

The multi-axle crane, also known as an all-terrain crane, is a complex machine that combines the mobility of a truck-mounted crane with the maneuverability of a rough-terrain crane. Its versatility makes it ideal for multi-use job sites, and it is typically equipped with a four-wheel drive (4WD) system powered by one or two engines. However, the handling maneuverability and load distribution of this crane present complicated challenges [1]. The multi-axle crane is a system that comprises a mounted crane, steering, and suspension systems. The performance of the steering system is a critical factor in evaluating the overall performance of the crane, particularly in terms of maneuverability and load distribution [2].

The Ackermann steering principle, commonly employed in multi-axle crane steering systems, achieves dynamic stability by utilizing different wheels based on driving speed. However, this strategy alone does not fully consider dynamic states such as lateral velocity and yaw rate, which are crucial for accurate path tracking [3]. As a result, numerous studies have sought to improve stability. A multi-axle crane has high inertia, which slows the dynamic response during steering, and it tends to introduce a time delay. A fast driving performance is required to ensure work efficiency and driving stability [4]. Therefore, driving stability at high speed, low-speed maneuverability, and accurate path tracking determined by steering performance are vital [5].

Various research efforts have focused on improving steering performance by optimizing the steering angle using conventional Ackermann principles and controllers such as Proportional-integral-derivative (PID), Linear Quadratic Regulator (LQR), Model Predictive Control (MPC), and other robust controllers. These approaches have provided insights into enhancing the steering efficiency of multi-axle cranes. However, this work takes a novel approach by developing a controller with an optimized weight for the steering input, ensuring accurate path tracking. This innovative approach, combined with the use of the Smell Agent Optimization (SAO) technique, sets this research apart and significantly enhances path-tracking performance under different driving speed conditions. SAO is a relatively new algorithm that was developed by Salawudeen et al. [6]. It mimics the behavior of an agent trying to identify the source of smell and has proven effective in path-tracking problems. It has been applied to several areas, such as the discrete Capacitated Vehicle Routing Problem for solid waste management [7], the frequency stabilization problem of an interconnected micro-grid [8–10], and speed control of the Direct Current (DC) motor [6,11], sizing of hybrid renewable energy systems [12], artificial intelligence, and image processing [13–15] etc. All these applications have shown the remarkable performance of SAO when compared to other meta-heuristic algorithms.

MPC has demonstrated a remarkable ability in handling inherent process delays by incorporating time delay models. MPC can also consider actuator limitations as

constraints and has preview capability, making it suitable for path-tracking control [16–18]. Moreover, MPC has proven to be successful in several applications, such as process optimization and control [19], energy management [20], vehicle convoy management [21], power electronics [22], Electric grid [23], and robotics [24], amongst several others. In this research, an Optimized MPC was designed to improve steering efficiency, dynamic stability, and path-tracking performance. The steering input weighting factor was optimized using the SAO to secure efficient path-tracking performance for any driving speed condition. Moreover, since MPC tuning accounts for a significant part of the costs associated with MPC projects, there have been efforts to tune and optimize MPC deployment automatically [25,26].

Recent advancements in embedded model predictive control for torque distribution optimization in electric vehicles have demonstrated that low computational burden strategies can be successfully combined with robust constraint handling to achieve real-time control performance [27]. In a similar vein, real-time nonlinear MPC strategies developed for distributed drive electric vehicles have underscored the importance of addressing dynamic nonlinearities and time delay effects—a challenge that is directly relevant to enhancing the steering control of multi-axle cranes [28].

Another challenge in the multi-axle crane domain is the limited availability of simulation, experimental, and implementation methods [29]. A simulation model was developed using AnyLogic software to address this issue. AnyLogic enables the integration of agent-based modeling, discrete event modeling, and system dynamics modeling, resulting in highly valid models [30,31]. The software's comprehensive libraries, including the road traffic network library, proved particularly suitable for this research. Additionally, AnyLogic provides the capability to simulate models in both 2D and 3D animation, which was utilized to visually analyze the multi-axle crane's behavior on the road and evaluate the impact of the designed steering control approach on the vehicle. The main contributions of this work are as follows:

(i) The development of an Optimal MPC (O-MPC) specifically tailored for multi-axle cranes, which ensures steering efficiency, dynamic stability, and improved path-tracking performance.

(ii) The introduction of a novel approach for optimizing the steering input weighting factor by employing SAO algorithm, which adjusts the MPC's control efforts based on driving speeds.

(iii) Developing a 3D simulation model using AnyLogic software allows for visual interpretation and validation of the improved performance, thereby providing a fairly realistic assessment of the system behavior under different speeds.

The paper is organized as follows. The next section presents some background on related works. Section 3 presents the methodology adopted for this work. The results and discussions are presented in section 4, and the paper is concluded in section 5, with some recommendations for further work.

2. Related works

This section provides an in-depth review of research on steering systems for multi-axle cranes, addressing challenges such as dynamic stability, steering efficiency, and path-tracking accuracy. The review consolidates key contributions, highlights gaps in the literature, and positions the current research within this context.

Du et. al. [5] developed an electro-hydraulic servo steering control for a 7-axle crane using a PID controller. Co-simulations with AMESim and ADAMS showed improvements in dynamic steering performance and tracking response, particularly on the seventh axle. Notably, the study suggested extending these improvements to additional axles.

Further advancing this area, a study [32], implemented an LQR-based steering controller with an adaptive weighting mechanism for all terrain. The aim was to enhance steering efficiency and dynamic stability. While the results showed a reduced yaw rate and improved efficiency during single-lane changes, this improvement came at the cost of higher mechanical steering effort, negatively affecting path tracking. Similarly, a hierarchical controller [33] for an 8-axle vehicle improved lateral stability and reduced tire wear during lane changes. However, this approach focused more on stability, compromising maneuverability. Jagirdar et al. [34] evaluated different steering strategies for a 4-axle vehicle, focusing on

handling improvements at various speeds. Although handling parameters were experimentally validated, the study's lack of attention to path tracking limited its overall contribution to steering control strategies.

Several MPC-based approaches have also emerged. One study [2] integrated a path-tracking controller into the MPC framework for optimizing steering angles, showing enhanced driving stability. However, increased mechanical effort across all speeds indicated room for further improvement in path tracking. Another MPC-based control approach to assist in steering an all-terrain crane, aiming to enhance steering efficiency and driving stability, was designed in [3]. The controller used a linear crane model and selected a wheel with the largest angular force among unused wheels in the Ackermann steering strategy to assist the driver. Simulations showed improved dynamic stability and steering efficiency, but the approach could not guarantee efficient path tracking. Similarly, [35] developed another MPC-based control system focusing on steering efficiency for a 5-axle crane, which improved efficiency but failed to ensure dynamic stability and accurate path tracking at higher speeds.

Oh et al. [4] investigated a Model Predictive Control (MPC) technique to optimize the steering angle to reduce the turning radius of all-terrain cranes. The control strategy, based on an error dynamic model from a linearized bicycle model, revealed that while the vehicle speed did not influence the optimized steering angle, the minimum turning radius decreased with lower speeds. However, the primary focus on improving maneuverability led to a trade-off in path-tracking performance. Recent advances in embedded model predictive control for torque distribution optimization in electric vehicles have demonstrated that low computational burden strategies—enabled by efficient constraint-handling techniques—can significantly improve real-time control performance. Such methodologies offer promising avenues for enhancing the multi-axle crane steering systems' dynamic response and stability [27]. Similarly, innovative nonlinear MPC approaches developed for yaw motion optimization in distributed drive electric vehicles have demonstrated the effectiveness of adaptive predictive horizons and robust initialization methods in managing system nonlinearities and constraints. These developments underline the potential for applying similar techniques to achieve more accurate path tracking and superior dynamic stability in multi-axle crane steering control [28].

Other works focused on using LQR control. In [34], an LQR controller was combined with a Recursive Least Squares (RLS) algorithm to enhance driving stability, reducing lateral velocity and yaw rate but sacrificing steering efficiency on curved paths. Milani et al. [36] took a different approach by applying a Quantum Particle Swarm Optimization (QPSO)-tuned LQR controller for heavy articulated vehicles. The work achieved notable improvements in low-speed maneuverability and high-speed stability, but path tracking was not adequately addressed.

On a different note, an adaptive fuzzy PID steering control system [37], validated for low-speed performance, demonstrated improvements in path tracking but lacked validation at higher speeds. A genetic algorithm-based steering optimization approach [21,38] was also introduced for multi-axle vehicles, achieving a reduced turning radius without addressing path-tracking or stability considerations.

Recent years have seen advanced control strategies applied to autonomous and heavy vehicles to improve stability and robustness under challenging conditions. Adaptive sliding mode control (SMC) methods, for instance, have demonstrated strong resilience to model uncertainties in vehicle dynamics. Norouzi et al. designed an adaptive SMC for a four-wheel-steering autonomous vehicle, achieving accurate orientation and position tracking despite parameter variations [39]. Non-singular terminal SMC variants can even ensure finite-time convergence of trajectory tracking errors under uncertain nonlinear dynamics. Meanwhile, fuzzy logic-based controllers offer effective output-feedback solutions without requiring full-state measurement. Nguyen et al. developed a fuzzy static output-feedback control for autonomous vehicle path-following that improved transient tracking performance [40]. Similarly, a Takagi–Sugeno fuzzy observer has been applied to a heavy truck–trailer system to handle unmodeled nonlinearities, enabling stable tracking with only output feedback [41]. These adaptive and fuzzy control schemes significantly enhance robustness against disturbances, model uncertainties, and actuator limits in heavy-duty vehicle applications.

Researchers have also addressed real-world constraints such as limited communication and cyber-attacks through event-triggered and resilient control frameworks. Ding et al. (2024) proposed an adaptive memory event-triggered

output-feedback controller for an autonomous heavy truck's lane-keeping system that co-designs the trigger and control law to guarantee finite-time stability while mitigating rollover risk [42]. This strategy reduces communication updates while ensuring the truck maintains its trajectory within prescribed performance bounds. To counteract malicious disruptions, Guo and Xu formulated an observer-based SMC for connected vehicles that preserves string stability even under denial-of-service attacks blocking inter-vehicle communication [43]. In a comprehensive approach, Mohammed et al. introduced a distributed event-triggered interval type-2 fuzzy SMC scheme that detects and tolerates multiple cyber-attacks in connected autonomous vehicle networks, effectively safeguarding platooning heavy trucks against network-borne disturbances [44]. These developments underscore the trend toward controllers that handle uncertainties and nonlinearities and maintain performance under sporadic updates and hostile conditions, which is crucial for next-generation autonomous and heavy vehicle systems.

It is evident that a lot of work has been done on the multi-axle crane steering system using the conventional Ackermann, skid steering and controllers such as PID, MPC, LQR, and Robust controllers. However, most of the works done tried to focus on solving either one or a combination of the problems of dynamic stability, steering efficiency (maneuverability), or path tracking performance (at high or low driving speed). Developing an optimal steering system that employs MPC with an SAO-based weighting factor, which considers the problem of dynamic stability, steering efficiency, and path tracking for better steering performance in varying driving speed conditions, is the motivation for this study.

3. Methodology

This section presents the methodology for developing multi-axle crane steering control using an optimized MPC. The methodology involves using a computer system with the following specifications: 6GB RAM, 500GB HDD, Intel® Core™ i3 CPU M30 @ 2.13GHz processor, AnyLogic software, and MATLAB/Simulink R2019b.

3.1 Simplified crane model

The representation of the 5-axle crane is given by a simplified linearized bicycle model, a single wheel at the center is made to represent the wheels at the right and left axles. The linear model was derived based on the assumption that the wheel load was constant, the slip angle was small, aerodynamic forces were not considered, and there was no braking or accelerating force. Fig 1 shows the simplified bicycle model in the x-y plane.

From the simplified bicycle model in Fig 1, the lateral crane dynamics can be described in terms of lateral translational motion and yaw rotation by applying Newton's second law in the y-axis:

$$mv\left(\psi + \dot{\beta}\right) = F_{y,1} + F_{y,2} + F_{y,3} + F_{y,4} + F_{y,5} \tag{1}$$

$$I\ddot{\psi} = L_1F_{y,1} + L_2F_{y,2} - L_3F_{y,3} - L_4F_{y,4} - L_5F_{y,5} \tag{2}$$

Where L_i and $F_{y,i}$ $(i = 1 - 5)$ represent the distance between mass center and i th axle, and the i th axle lateral tire force respectively, m is mass of crane, v is longitudinal velocity, β is the sideslip angle, and I is the moment of inertia of the crane. To simplify the model, it was assumed that the longitudinal velocity of the crane was kept constant and lateral tire force is proportional to small slip angle.

State and input variables describing the crane dynamics are given as:

$$x = \begin{bmatrix} z & \dot{z} & \psi & \dot{\psi} \end{bmatrix}^T \tag{3}$$

$$u = \begin{bmatrix} \delta_1 & \delta_2 & \delta_3 & \delta_4 & \delta_5 \end{bmatrix}^T \tag{4}$$

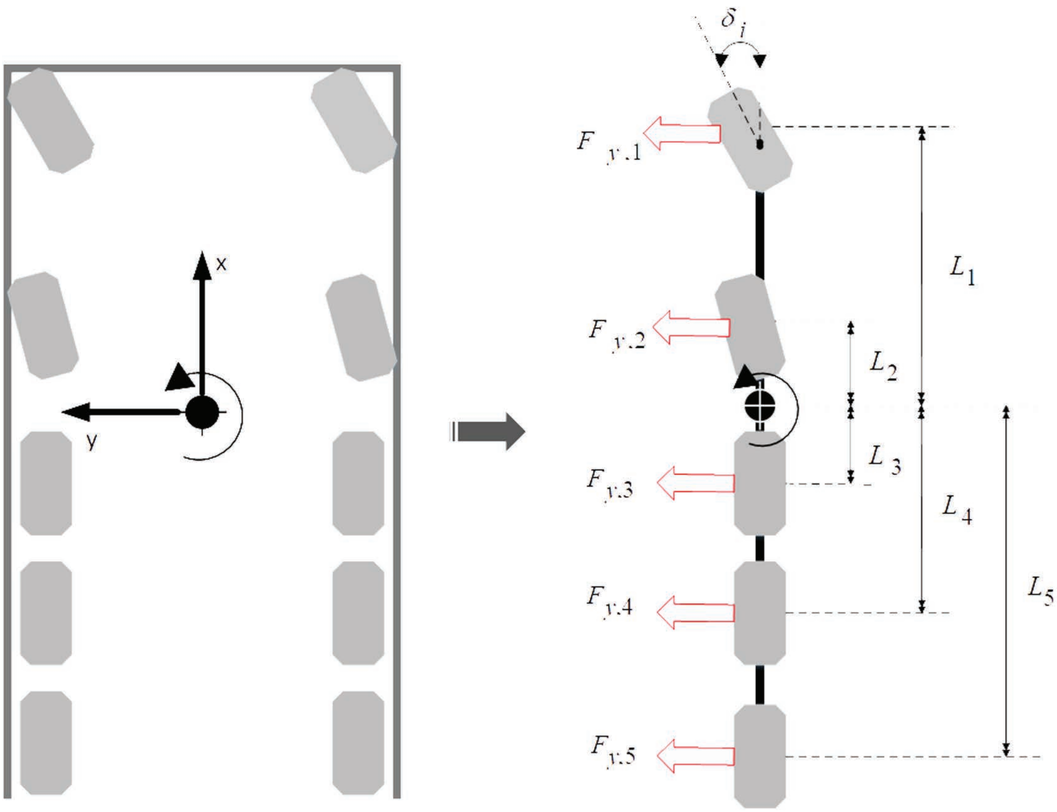

Fig 1. Simplified Bicycle Model in x-y Plane.

https://doi.org/10.1371/journal.pone.0324720.g001

Where z is the lateral position of the crane, $\dot{z}$is the lateral velocity, ψ is the yaw angle, and $\dot{\psi}$ is the yaw rate. While $\delta_{(i=1-5)}$ represents the steering angle of the wheel at ith axle.

The representation of the simplified linear bicycle model in state space form using the state and input variables is given as:

$$\dot{x} = Ax + Bu \tag{5}$$

Where:

$$A = \begin{bmatrix} 0 & 1 & 0 & 0 \\ 0 & -\frac{2}{mv_x}\sum_{i=1}^{5} C_i & 0 & \frac{2}{mv_x}\left(-\sum_{i=1}^{2} L_iC_i + \sum_{i=3}^{5} L_iC_i\right) - v_x \\ 0 & 0 & 0 & 1 \\ 0 & -\frac{2}{I_zv_x}\left(-\sum_{i=1}^{2} L_iC_i + \sum_{i=3}^{5} L_iC_i\right) & 0 & -\frac{2}{I_zv_x}\sum_{i=1}^{5} L_i^2C_i \end{bmatrix} \tag{6}$$

$$B = \begin{bmatrix} 0 & 0 & 0 & 0 & 0 \\ \frac{2C_1}{m} & \frac{2C_2}{m} & \frac{2C_3}{m} & \frac{2C_4}{m} & \frac{2C_5}{m} \\ 0 & 0 & 0 & 0 & 0 \\ \frac{2L_1C_1}{I_z} & \frac{2L_2C_2}{I_z} & -\frac{2L_3C_3}{I_z} & -\frac{2L_4C_4}{I_z} & -\frac{2L_5C_5}{I_z} \end{bmatrix} \tag{7}$$

Where L_i represent the distance between mass center and ith axle, C_i is the ith axle cornering stiffness, m is mass of crane, v_x is longitudinal velocity, I_z is the crane's moment of inertia.

In order to design a steering controller, a dynamic model of the crane with the state variables in terms of error with respect to the road is required. The multi-axle crane dynamic model can be expressed in terms of error in state space with respect to lateral error described by (8) and yaw angle error which is described by (9).

$$e_1 = z - z_{des} \tag{8}$$

$$e_2 = \psi - \psi_{des} \tag{9}$$

Where e_1 is the lateral error with z as the vehicle's lateral velocity and z_{des} as the desired value. e_2 is the yaw angle error with ψ as the yaw angle and ψ_{des} as the desired value. The representation of the dynamic model in terms of error in state space is given as:

$$\dot{e} = A_e e + B_e u \tag{10}$$

$$\Delta y = C_e e \tag{11}$$

Where:

$$e = \begin{bmatrix} e_1 & \dot{e}_1 & e_2 & \dot{e}_2 \end{bmatrix}^T \tag{12}$$

$$A_e = \begin{bmatrix} 0 & 1 & 0 & 0 \\ 0 & -\frac{2}{mv_x}\sum_{i=1}^{5} C_i & \frac{2}{m}\sum_{i=1}^{5} C_i & \frac{2}{mv_x}\left(-\sum_{i=1}^{2} L_iC_i + \sum_{i=3}^{5} L_iC_i\right) \\ 0 & 0 & 0 & 1 \\ 0 & \frac{2}{I_zv_x}\left(-\sum_{i=1}^{2} L_iC_i + \sum_{i=3}^{5} L_iC_i\right) & \frac{2}{I_z}\left(\sum_{i=1}^{2} L_iC_i + \sum_{i=3}^{5} L_iC_i\right) & \frac{2}{I_zv_x}\sum_{i=1}^{5} L_i^2C_i \end{bmatrix} \tag{13}$$

$$B_e = \begin{bmatrix} 0 & 0 & 0 & 0 & 0 \\ \frac{2C_1}{m} & \frac{2C_2}{m} & \frac{2C_3}{m} & \frac{2C_4}{m} & \frac{2C_5}{m} \\ 0 & 0 & 0 & 0 & 0 \\ \frac{2L_1C_1}{I_z} & \frac{2L_2C_2}{I_z} & -\frac{2L_3C_3}{I_z} & -\frac{2L_4C_4}{I_z} & -\frac{2L_5C_5}{I_z} \end{bmatrix} \tag{14}$$

$$C_e = [1\ 0\ 1\ 0] \tag{15}$$

And u is as defined in equation (4).

3.2 Model predictive control

Model Predictive Control (MPC) relies on predictions and estimates of the system's current state to optimize a control cost function over a predefined time horizon. The resulting solution consists of a sequence of present and future control actions to drive the system toward optimal performance based on the chosen objective function. The first control action in this sequence is applied immediately, while the process is repeated iteratively. Recent advancements in MPC primarily

utilize the state-space formulation [45,46], though implementation results remain comparable across different approaches. Notably, the velocity-based state-space formulation provides key advantages, such as ensuring offset-free control [46].

In this study, we employ a discrete state-space model with an augmented velocity formulation, as represented in (16) and (17).

$$x(k+1) = A_m x_m(k) + B_m u(k)$$

$$y(k) = C_m x_m(k) \tag{16}$$

$$y(k+1) = Ax(k) + B\Delta u(k)$$

$$y(k) = Cx(k) \tag{17}$$

The state space matrices denoted, $A \in \mathbb{R}^{n\times n}$, $B \in \mathbb{R}^{n\times m}$, and $C \in \mathbb{R}^{p\times n}$are defined in (18), *0* and *I* are matrices of zeros and ones with appropriate dimensions.

$$A = \begin{bmatrix} A_m & 0_p^T \\ C_m A_m & I_p \end{bmatrix},\ \begin{bmatrix} B_m \\ C_m B_m \end{bmatrix},\ C = \begin{bmatrix} 0_{n\times p} & I_p \end{bmatrix} \tag{18}$$

The optimization cost function in MPC is designed to penalize both tracking error and control effort variations, as expressed in Equation (19). However, we focus the penalty solely on the manipulated variable, allowing for more precise tuning.

$$J = \sum_{i=1}^{P} \|r(k+i) - y(k+i)\|_Q^2 + \sum_{i=1}^{M} \|\Delta u(k+i)\|_R^2 \tag{19}$$

The prediction horizon, P, is selected to adequately capture process dynamics. For the control horizon, previous studies have shown that a value between 3 and 5 is sufficient [47]. Hence, a control horizon of $M = 3$ is used in this study. The diagonal matrices Q and R are positive definite weighting matrices of appropriate dimensions. Increasing beyond five typically offers no additional benefits. By predefining these parameters, the number of tuning variables is reduced from four to one, thereby simplifying the controller design.

3.3 Design of O-MPC steering system

This section discusses the design of the MPC with an optimized weighting factor based on a metaheuristic approach. This weighting factor uses the SAO algorithm and is applied in the MPC controller as the control input weight.

3.3.1 Weighing factor cost formulation. The driver's intention can be described using components of the steering input, such as the steering angle and its rate of change. In order to ensure dynamic stability and to obtain the desired crane states together with the driver's intention, a weighting factor rule for the MPC was adopted. The weight on the input in the MPC cost function, represented by R, will be determined using the weighting factor. This will compute the optimal steering angle of the wheel using the steering angle input and rate of change. The weighing factor is presented in (20) and formulated into an objective function to obtain an optimized weight for the controller input.

$$R(i) = |m_i\dot{\delta}_1 + n_i\delta_1 + p_i| \tag{20}$$

Where δ_1 is the is the steering angle at the 1st axle and $\dot{\delta}_1$ is the steering angle rate. The objective of the optimization problem is to minimize the weight by optimizing the weighting factor. The objective function is given as:

$$f(x) = \min \sum_{i=1}^{N} R(i) = \min \sum_{i=1}^{N} |m_i \dot{\delta}_1 + n_i \delta_1 + p_i| \quad (21)$$

The optimum solution is determined subject to the constraints:

$$0 \leq m_i \leq 2000 \quad (22)$$

$$0 \leq n_i \leq 1000 \quad (23)$$

$$0 \leq p_i \leq 4000 \quad (24)$$

Where m_i, n_i, and p_i are the parameters for computing the weighing factor (at time instance i) which are positive coefficients that must be optimized to minimize the objective function.

The SAO algorithm is employed in this study to optimize the weighting factor parameters (m, $n$and$p$). The flowchart for implementing the smell agent optimization is shown in Fig 2.

At the beginning stage, the initial population of size N is generated, and the values of the weighing factor parameters (m, n, and p) were randomly selected based on the constraints given in (22), (23), and (24). The SAO's selection process then searches for the best combination of the parameters that will obtain the optimal solution. In search of the objective function's optimum, the algorithm's three modes (sniffing, trailing, and random mode) are used.

The parameters in the search space follow the trail of the best smell molecule. The objective function in (21) serves as an evaluation criterion for the optimality of the results obtained by SAO; it is optimized until the termination criteria are reached. Fig 3 gives the flowchart for evaluating the cost function using SAO.

The SAO parameters used in this study are presented in Table 1.

3.3.2 Designing the MPC with the developed SAO weighting factor. The steering system of the multi-axle crane has the 1st and 2nd axles mechanically linked together. Hence, the driver's steering input determines the wheel angle of the 2nd axle. Furthermore, the conventional steering system uses the Ackerman strategy to determine the wheel angle of the 3rd, 4th, and 5th axles. As such, the MPC controller with SAO weighting factor is designed to compute the optimal steering angle of the multi-axle crane's 3rd, 4th, and 5th axles. It takes into consideration the defined weight and constraints. The schematic model of the O-MPC is depicted in Fig 4.

The MPC was created using the defined model in (10) and (11) with a sample time of 0.1, prediction and control horizons of $P = 20$ and $M = 3$, respectively. The manipulated variable weight was set to be a time-varying function in Simulink, and it was defined to be the developed SAO-based weighing factor. The following constraints on the manipulated variable u and its rate Δuwere used:

$$-\frac{\pi}{6} \leq u \leq \frac{\pi}{6} \quad (25)$$

$$-\frac{\pi}{12} \leq \Delta u \leq \frac{\pi}{12} \quad (26)$$

Equation (25) represents the steering angle constraint, which is based on the physical limitations of the wheel. Since this is not directly in the MPC formulation, any values of u obtained are checked to ensure that they respect the constraints. Equation (26) represents the constraint on the rate of change of the steering angle. These constraints play a crucial role in

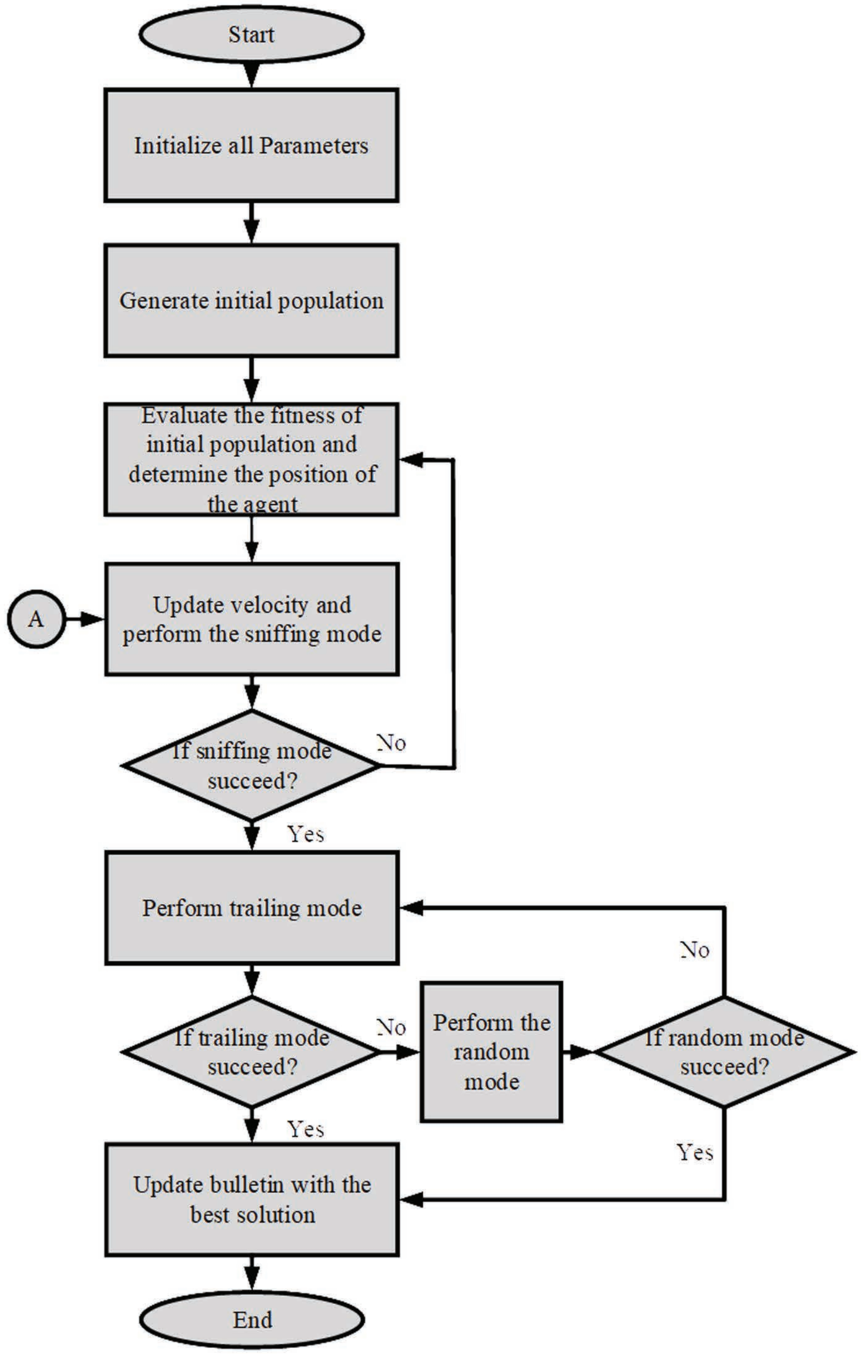

Fig 2. Flow Chart of SAO.

https://doi.org/10.1371/journal.pone.0324720.g002

the control system, ensuring that the steering angle and its rate of change remain within acceptable limits. The flowchart in Fig 5 provides a detailed overview of the MPC implementation with the SAO weighting factor.

The process begins with defining the plant model. Then, the controller generation parameters, such as prediction horizon, control horizon, and sample time, are defined. A performance criterion is then defined for the MPC, where the

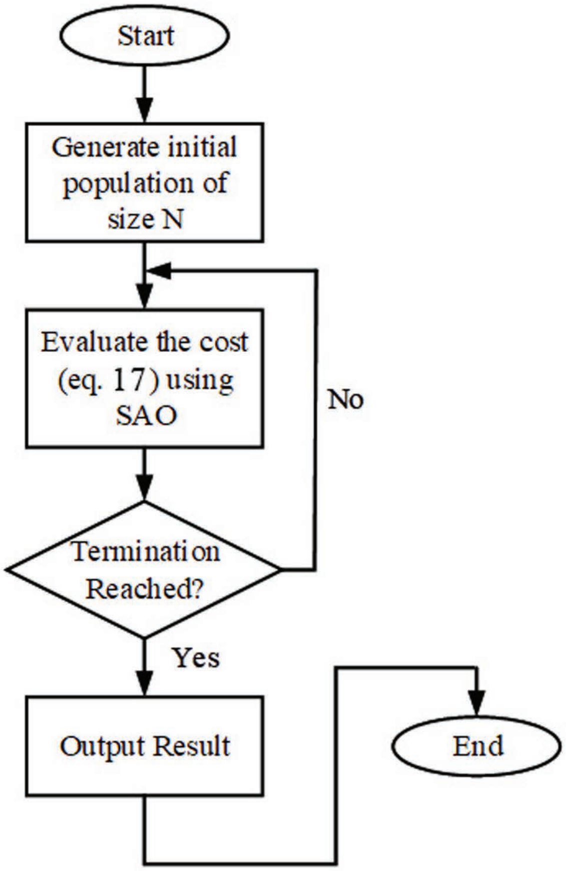

Fig 3. Flow Chart of Weighing Factor Cost Evaluation.

https://doi.org/10.1371/journal.pone.0324720.g003

Table 1. SAO Parameters.

Parameter	Value
Temperature, T (K)	3
Boltzmann's Constant, k (JK^{-1})	1.38×10^{-23}
Population (Molecules), N	50
Dimension, D	3
Iteration, Itr	10
Step Movement, SM	2.5

https://doi.org/10.1371/journal.pone.0324720.t001

weighting factor is used as the input weight. This weighting factor is optimized using the Smell Agent Optimization (SAO) technique based on the formulated cost function outlined in (21). The MPC then solves the quadratic programming (QP) problem to determine the optimal control inputs.

The process initiates with the application of the first control input to the plant, which is part of the sequence of optimal inputs obtained. At each sampling instant, the QP optimization is repeated until the termination condition is met. This iterative process ensures that the control system continuously and adaptively adjusts the control inputs based on the plant's current state.

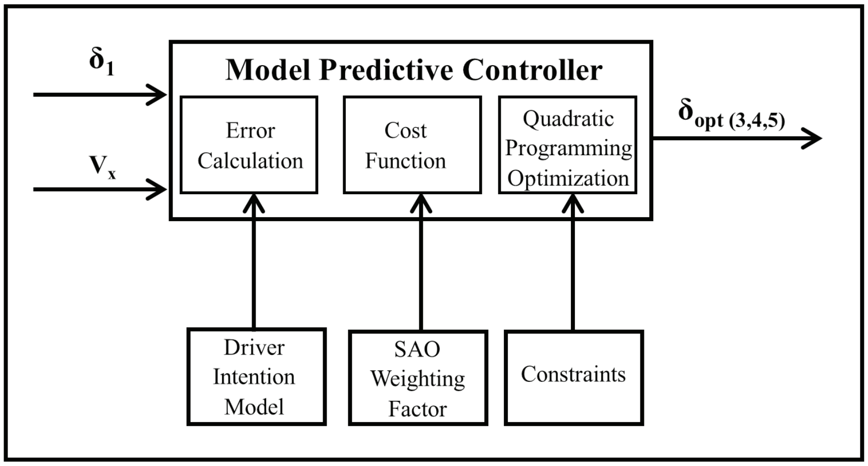

Fig 4. O-MPC schematic model.

https://doi.org/10.1371/journal.pone.0324720.g004

3.3.3 Developing the Driver Intention Model. The driver intention model, which provides crucial feedback to the Optimized MPC is described by (27) and (28):

$$e_{lateral} = -k_{dt,\ lateral} \frac{(L_p - L_1)\tan(\delta_1)}{v_x} \tag{27}$$

$$e_{yaw} = -k_{dt,\ yaw} \frac{\delta_1}{v_x} \tag{28}$$

Where $e_{lateral}$ and e_{yaw} are the lateral error and yaw angle error respectively, $k_{di,lateral}$ and $k_{di,yaw}$ are proportional constants for the driver's intention. δ_1 is the steering angle at the 1st axle, L_p is the preview distance from the crane mass center to the preview point at which the driver gets the information of the path, and L_1 is the distance between the crane mass center and the 1st axle.

Unlike traditional error-based feedback, as defined in (13) and (14), the driver intention model significantly enhances the MPC's performance. It offers feedback in terms of expected errors relative to the road path, reflecting the driver's intentions and helping eliminate conflicts between the driver's steering input and that of the controller. As a result, the optimized MPC can compute the optimal steering angles for the 3rd, 4th, and 5th axles based solely on the driver's intention. The parameters used for the driver intention model are
$k_{di,lateral} = 25$ and $k_{di,yaw} = 22$.

3.4 3D simulation model

A comprehensive co-simulation approach, integrating Simulink and AnyLogic software, was utilized to develop the 3D simulation model for the proposed optimized MPC with the multi-axle crane model. This approach ensures a thorough and detailed assessment of the MPC's performance in different driving speed conditions.

3.4.1 Simulation model using Simulink. In order to develop a proper representation of the vehicle dynamics, the linearized dynamic model of the multi-axle crane, given in state space and represented in equation (10), was used to design the Simulink model. The parameters of the multi-axle crane used were derived based on the following: General

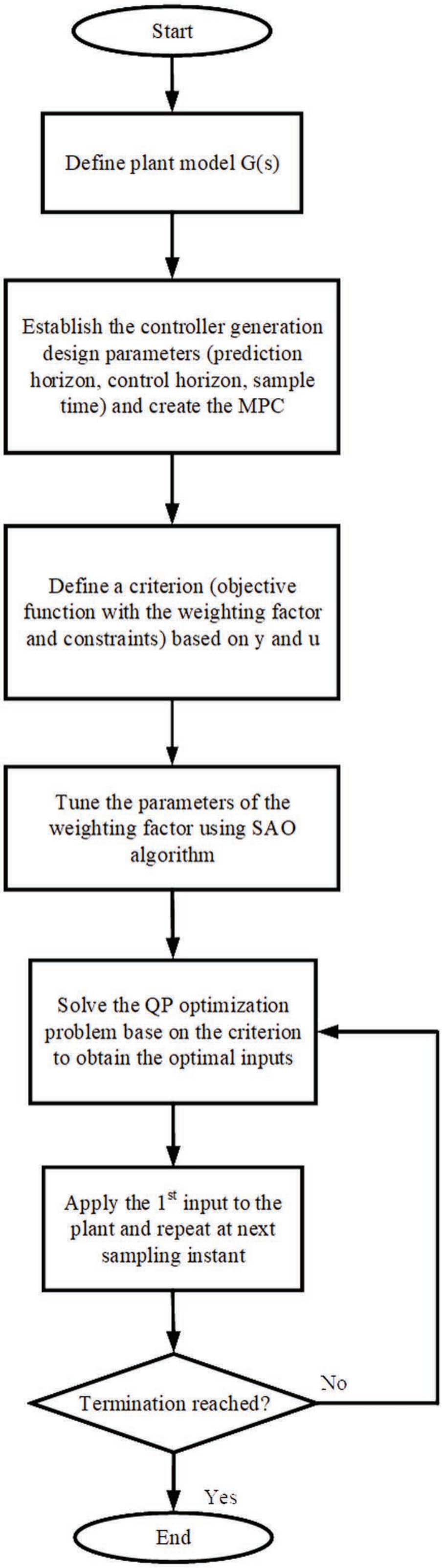

Fig 5. Flow Chart for the Implementation of O-MPC.

https://doi.org/10.1371/journal.pone.0324720.g005

Table 2. Multi-axle Crane Model Parameters.

Parameter	Value
**Mass, m (kg)**	60,000
**Moment of inertia, I (Kgm^2)**	1,110,000
**Cornering stiffness, C_i $i = 1 - 5$ (N/rad)**	300,000
**1st axle distance to mass center, L_1 (m)**	3.968
**2nd axle distance to mass center, L_2 (m)**	1.408
**3rd axle distance to mass center, L_3 (m)**	0.242
**4th axle distance to mass center, L_4 (m)**	2.052
**5th axle distance to mass center, L_5 (m)**	3.752

https://doi.org/10.1371/journal.pone.0324720.t002

on-road driving scenario, normal driving condition, and Pacekja magic formula was used as the tire model to derive the cornering stiffness. The parameters are given in Table 2. In this simplified formulation, the cornering stiffness among axles is assumed to be equal. This is because a small slip angle is assumed under normal driving conditions. In actual operating conditions, variations in stiffness among axles is expected, which could affect model accuracy. However, feedback control could cater for such inaccuracies.

The Optimized MPC with the multi-axle crane model was simulated on a curved road path. The driving scenario designer, part of the automated driving toolbox found in MATLAB/Simulink, was used to design the reference road path for the vehicle simulation and performance evaluation. It allows the user to design a wide range of road paths with different patterns and specifications. A radius of curvature and driving distance of 60 m and 900 m were used for the curved road path, respectively.

The closed-loop simulation was conducted using a driver model from the Vehicle Dynamics Blockset in MATLAB/Simulink. This driver model closes the loop between the reference road path and the actual vehicle path. It takes in the vehicle states, velocity, and reference road information as input to generate a steering angle for the first axle of the crane to track the path. The O-MPC computes the optimal steering angles for the 3rd, 4th, and 5th axles. Fig 6 shows the Schematic model of the Optimized MPC steering system.

Simulations were run for three scenarios at 25 km/h, 45 km/h, and 65 km/h, capturing the vehicle trajectory along a curved road path. The trajectory data was then exported to AnyLogic software for 3D simulation, allowing for a visual assessment of the multi-axle crane's behavior and the effectiveness of the Optimized MPC in different driving speed conditions.

3.4.2 Simulation model using AnyLogic. To perform a 3D simulation in AnyLogic, the multi-axle crane was initially modeled in 3D using the Agent Library palette. This process involved creating a detailed 3D representation of the crane, including components such as the axles, wheels, and chassis. The Agent Library palette in AnyLogic provided the tools to define the crane's physical properties, behavior, and interactions within the simulation environment.

Following the crane modeling, a space markup was performed on the graphical editor to design the road network. Using the Road Traffic Library, a curved road was created based on the waypoints obtained from the Simulink simulation. This design is depicted in Fig 7, showcasing the 3D representation of the curved road path.

The crane's movement along the road network was defined using a Flowchart in AnyLogic. This Flowchart, constructed by adding and connecting blocks from the library palette, executes the crane's behavior, including speed, direction, and environmental interaction. The actual trajectory data from the Simulink simulation was imported into AnyLogic to guide the crane's movement along the road path for three scenarios (25, 45, and 65 km/h). Fig 5 displays the Flowchart used for the simulation, highlighting the three main blocks: the **Car Source Block**, which generates and places the

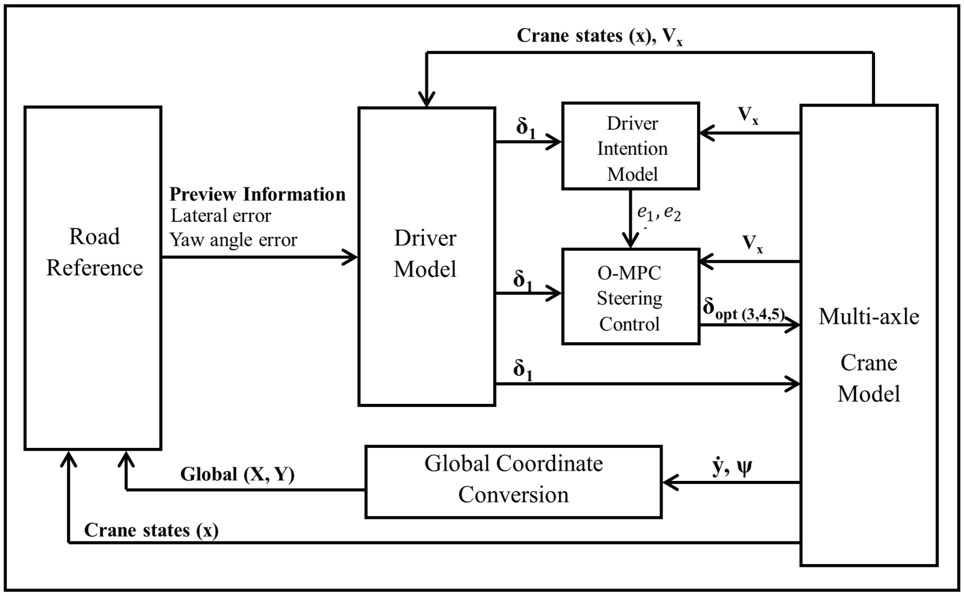

Fig 6. Schematic model of O-MPC steering system.

https://doi.org/10.1371/journal.pone.0324720.g006

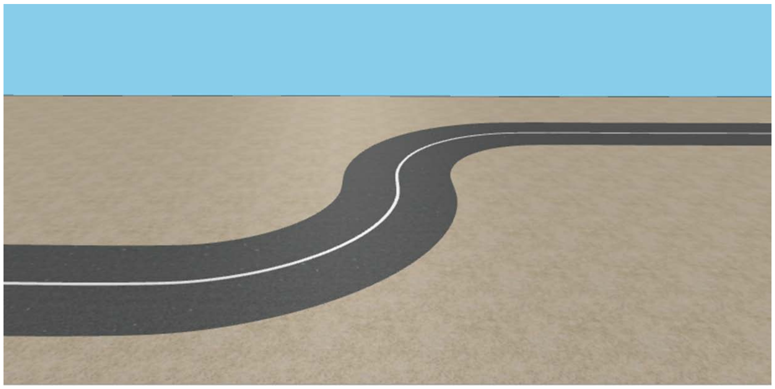

Fig 7. Curved road path.

https://doi.org/10.1371/journal.pone.0324720.g007

crane model; the **Car Move to Block**, which controls the crane's movement; and the **Car Dispose Block**, which signifies the end of the simulation. Fig 8 shows the AnyLogic flowchart of the multi-axle crane simulation for three scenarios.

The AnyLogic 3D window from the presentation palette was used to visualize the crane's movement and deviation from the desired curved road path in 3D. This feature provided a detailed view of the crane's performance along the road path, as illustrated in Fig 9.

4. Results and discussion

This section presents the results obtained from simulating the developed O-MPC steering system for the multi-axle crane, comparing its performance with the steering system developed by Oh and Seo [3], which is referred to as the benchmark in subsequent sections. The evaluation focuses on three key metrics: steering efficiency, dynamic stability, and path-tracking performance. The steering efficiency was obtained quantitatively using the formula given in equation (29). The

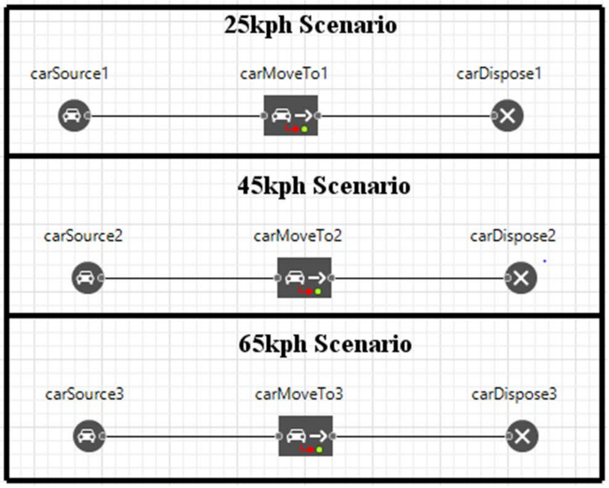

Fig 8. AnyLogic flowchart of multi-axle crane simulation for three scenarios.

https://doi.org/10.1371/journal.pone.0324720.g008

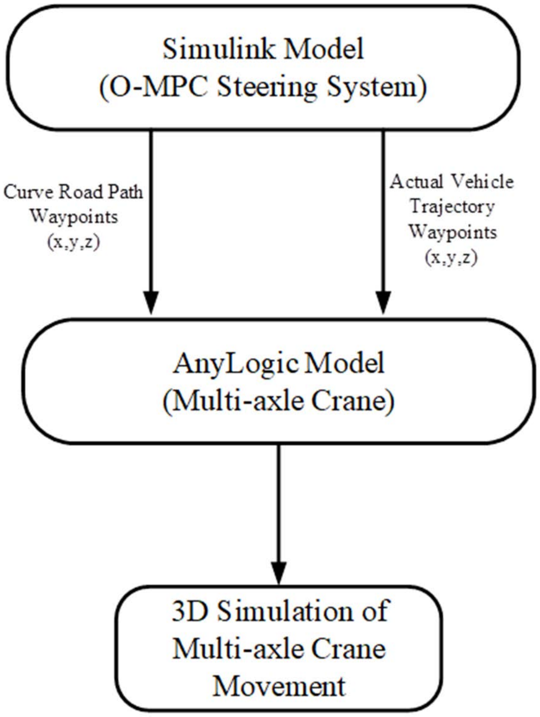

Fig 9. Framework for 3D Simulation of Multi-axle Crane.

https://doi.org/10.1371/journal.pone.0324720.g009

yaw rate of the multi-axle crane was used as a measure of dynamic stability, while lateral error and yaw angle error were used as measures of path-tracking performance.

$$S.E = \frac{D}{S_{effort}} \tag{29}$$

Where $S.E$ is the steering efficiency, D is the driving distance and S_{effort} is the driver's steering effort, which is expressed as:

$$S_{effort} = \int_{o}^{t} \left|\dot{\delta}_1\right| dt \tag{30}$$

With t as the driving time and $\dot{\delta}$ is the steering angle rate at the 1st axle. Simulations were conducted under three different driving speed scenarios: 25, 45, and 65 km/h to assess how the O-MPC system performs relative to the existing approach under these varied conditions.

4.1 Weighting factor optimization

The parameters of the weighting factor were optimized using the Smell Agent Optimization (SAO) method with the pre-defined optimization parameters listed in Table 1. The results of the optimized parameters for the three different driving speed scenarios are provided in Table 3.

The parameters in Table 4 were computed into the weighting factor and used for the simulation of the optimized MPC steering system. Table 4 presents the performance evaluation of SAO in optimizing the weighting factor cost function. Figs 10–12 show plots showing the optimization process for the three scenarios.

Figs 9–11 show that the algorithm effectively minimizes the weighting factor cost function across all three driving speed scenarios. However, the optimization process requires much iteration to achieve convergence. This high iteration count

Table 3. Optimal Values of Weighting Factor Parameters.

Parameter	25km/h Scenario	45km/h Scenario	65km/h Scenario
m	357.8	535.7	123.3
n	107.1	43.5	92.0
p	179.9	2406.3	839.8

https://doi.org/10.1371/journal.pone.0324720.t003

Table 4. Performance Evaluation of SAO on Weighting Factor Cost Function.

Scenarios	Cost	Time(s)
**25km/h Scenario**	1304.6	72936
**45km/h Scenario**	962.3	63108
**65km/h Scenario**	789.9	59652

https://doi.org/10.1371/journal.pone.0324720.t004

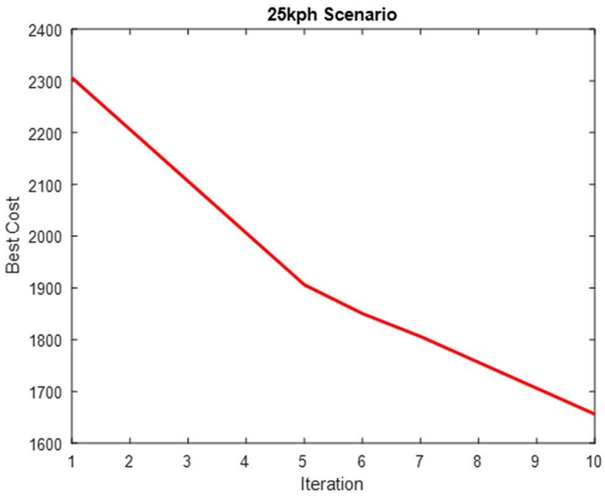

Fig 10. Weighting Factor Cost Optimization (25 km/h Scenario).

https://doi.org/10.1371/journal.pone.0324720.g010

is due to the model's complexity, as it must be optimized at each waypoint along the road while accounting for varying changes in road conditions, resulting in a computationally intensive process. This weighting factor optimization is solved offline and does not add to MPC's online computation requirements.

While this may seem to add to the already high computational requirements of m

4.2 Simulation results

The simulation results of the optimized MPC steering system were assessed with respect to steering efficiency, dynamic stability, and path-tracking performance, including lateral error and yaw angle error. The results were visualized through plots, which were compared with the replicated work of Oh and Seo [3] to highlight performance improvements. The following sections present the results in detail.

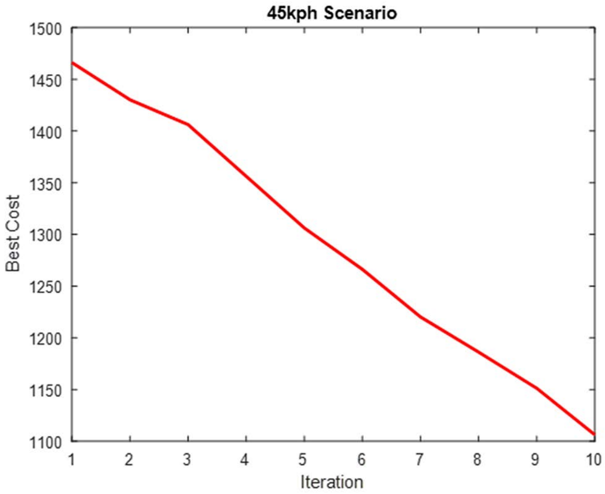

Fig 11. Weighting Factor Cost Optimization (45 km/h Scenario).

https://doi.org/10.1371/journal.pone.0324720.g011

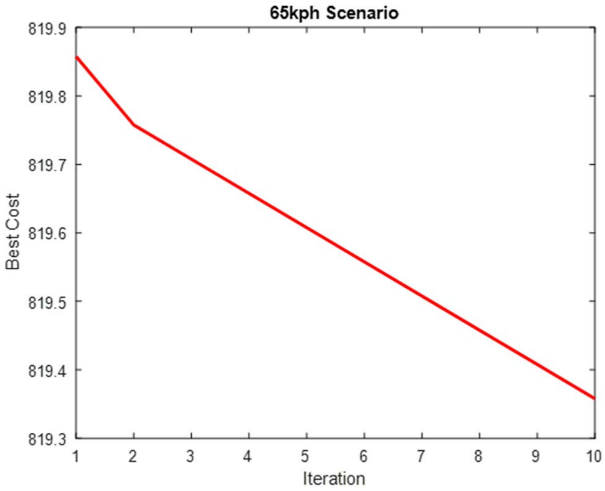

Fig 12. Weighting Factor Cost Optimization (65 km/h Scenario).

https://doi.org/10.1371/journal.pone.0324720.g012

4.2.1 Steering efficiency result. The driver's steering effort was evaluated based on the 1st axle steering angle. The results for the steering effort obtained in the three scenarios are given in Figs 13–15.

Figs 13–15 show that the O-MPC steering system significantly reduces the driver's steering effort compared to the existing scheme. Specifically, in the 25 km/h scenario, the steering effort decreased from 2.83 to 2.49; in the 45 km/h scenario, it reduced from 3.98 to 2.73; and in the 65 km/h scenario, it went down from 3.37 to 2.85. This demonstrates that the O-MPC steering system effectively calculates an optimal steering angle based on the driver's intentions, thereby reducing the effort required to manage the multi-axle crane across all three scenarios. These results where further evaluated to obtain the steering efficiency. The results are summarized in Table 5.

Table 5 presents the performance evaluation of the steering efficiency for the O-MPC and the existing scheme. It can be seen that the steering efficiency with the developed O-MPC steering system is increased compared to the existing

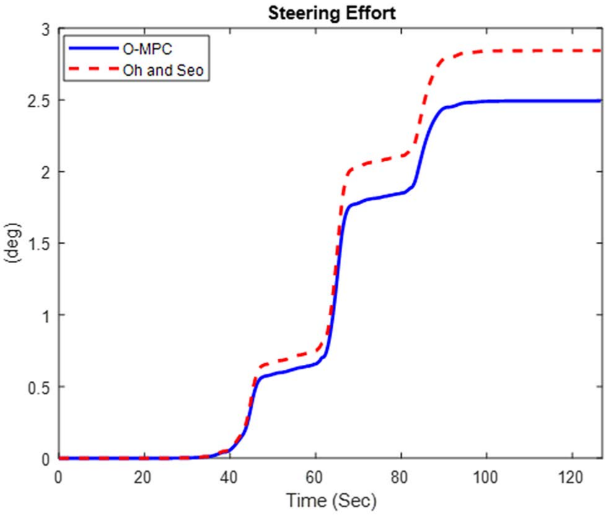

Fig 13. Steering Effort at 25 km/h.

https://doi.org/10.1371/journal.pone.0324720.g013

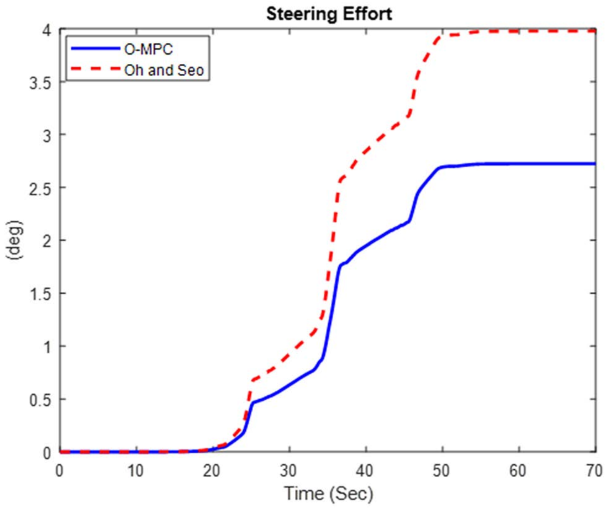

Fig 14. Steering Effort at 45 km/h.

https://doi.org/10.1371/journal.pone.0324720.g014

scheme. This is due to the fact that the O-MPC reduced the steering effort in all three scenarios. This shows that the O-MPC provides better steering efficiency in comparison to the existing scheme.

4.2.2 Dynamic stability result. Figs 16–18 show the multi-axle crane's yaw rate, which shows its dynamic stability for the three different scenarios.

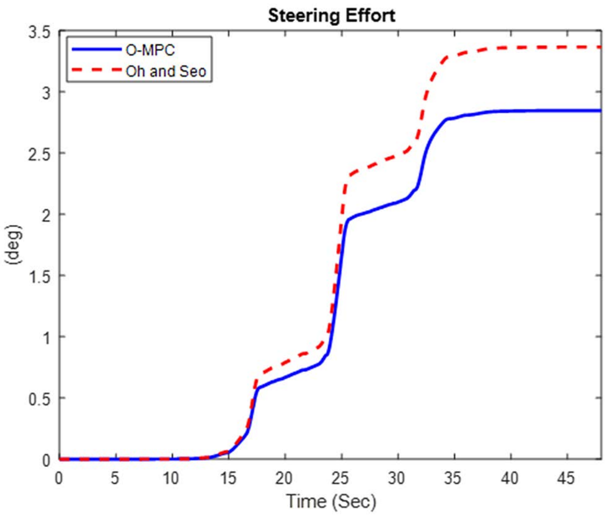

Fig 15. Steering Effort at 65 km/h.

https://doi.org/10.1371/journal.pone.0324720.g015

Table 5. Performance Evaluation of Steering Efficiency.

Scenario	25km/h	45km/h	65km/h	Unit
**O-MPC**	361	330	316	m/deg
**Benchmark**	317	226	267	m/deg

https://doi.org/10.1371/journal.pone.0324720.t005

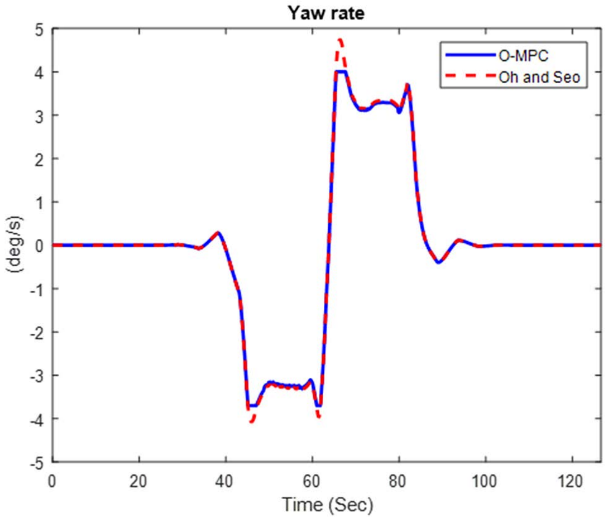

Fig 16. Yaw Rate of the Multi-axle Crane at 25 km/h.

https://doi.org/10.1371/journal.pone.0324720.g016

The analysis of Figs 16–18 shows that the yaw rate of the multi-axle crane when using the O-MPC steering system was reduced, and the oscillation was less when compared to the existing scheme for all three scenarios. This shows that the O-MPC provides improved dynamic stability, which can be attributed to the optimized weighting factor, which considers the changes in driving speed conditions, thereby providing an appropriate weight for the steering input, which reduces the yaw rate.

4.2.3 Tracking performance result. We obtained the tracking performance, given by the lateral error and yaw angle error, with a 3D representation of these results Figs 19–21 show the lateral error between the reference path (curve road path) and the center of mass of the multi-axle crane. The results show that the error was reduced in all three scenarios.

Figs 22–24 show the yaw angle error between the curved road path and the center of mass of the multi-axle crane

The results show that the yaw angle error was also reduced in all the scenarios. Hence, from the analysis of the results obtained for both the lateral and yaw angle errors, it can be concluded that the optimized weighting factor penalizes the

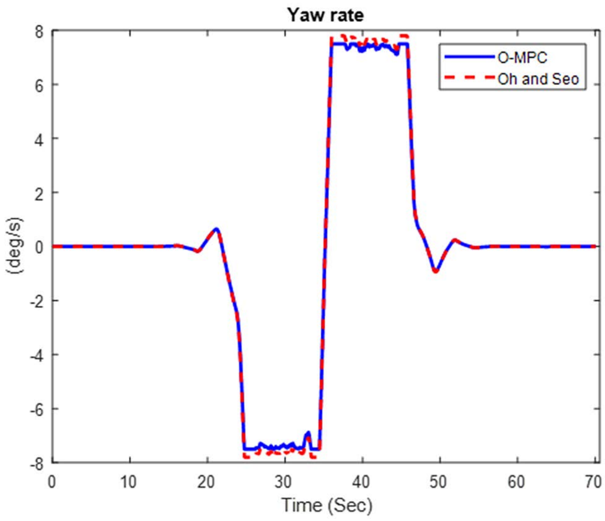

Fig 17. Yaw Rate of the Multi-axle Crane at 45 km/h.

https://doi.org/10.1371/journal.pone.0324720.g017

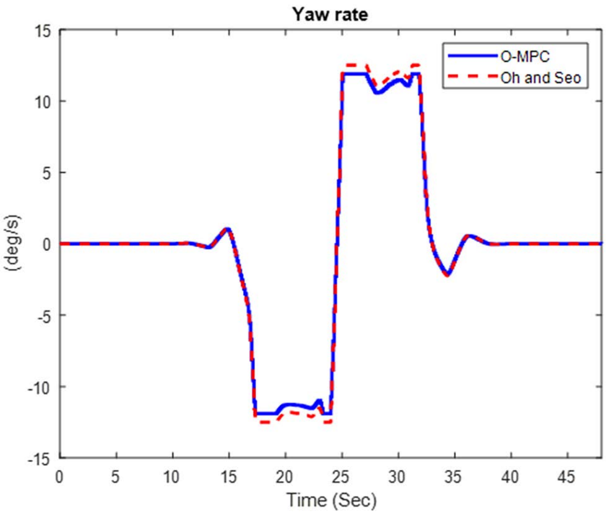

Fig 18. Yaw Rate of the Multi-axle Crane at 25 km/h.

https://doi.org/10.1371/journal.pone.0324720.g018

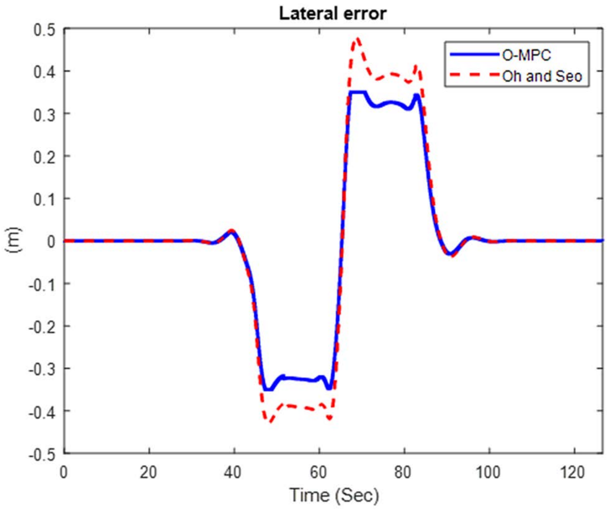

Fig 19. Lateral Error at 25 km/h.

https://doi.org/10.1371/journal.pone.0324720.g019

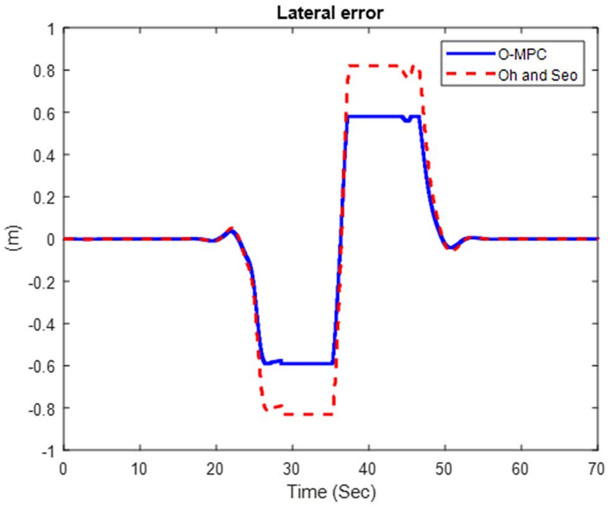

Fig 20. Lateral Error at 45 km/h.

https://doi.org/10.1371/journal.pone.0324720.g020

steering input appropriately based on the driving speed. This, in turn, provides an accurate steering angle that can track the reference path with higher accuracy than the existing scheme. This was further analyzed visually with the tracking results obtained in 3D, shown in Figs 25–27.

Figs 25–27 show the tracking results in a 3D simulation, which was used for further analysis and verification. This gave a visual interpretation of the tracking results obtained, and it can be seen from the results that the distance from the center of mass of the multi-axle crane to the reference path was reduced in all the scenarios. These tracking results showed that the O-MPC steering system was able to provide a better tracking performance regardless of the change in speed when compared to the existing scheme.

4.3 Comparative analysis

The performance of the O-MPC steering system is compared to the work of Oh and Seo [3], our benchmark, across three key metrics: steering efficiency, dynamic stability, and tracking performance. The comparison is based on data computed from the simulation plots, where the yaw rate and tracking results were evaluated using Root Mean Square (RMS) values. The results for the three different driving speed scenarios are presented in Tables 6 to 7, providing a detailed performance comparison between the O-MPC steering system and the previously developed system by Oh and Seo [3].

Tables 6–8 summarize the performance comparison between the developed O-MPC steering system and the system by Oh and Seo [3] across three different driving speed scenarios. The comparison reveals that the O-MPC steering

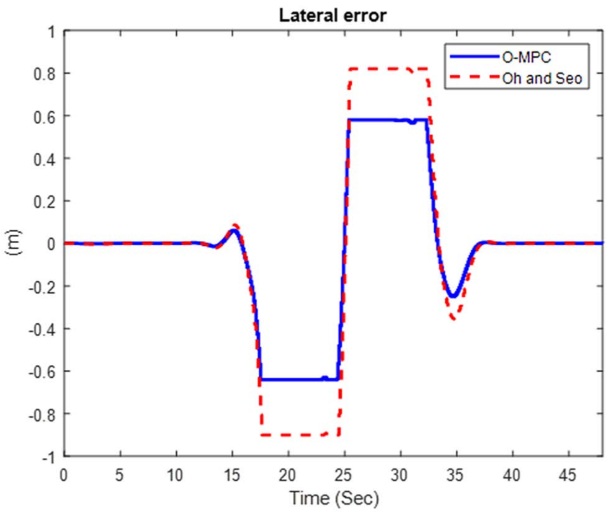

Fig 21. Lateral Error at 65 km/h.

https://doi.org/10.1371/journal.pone.0324720.g021

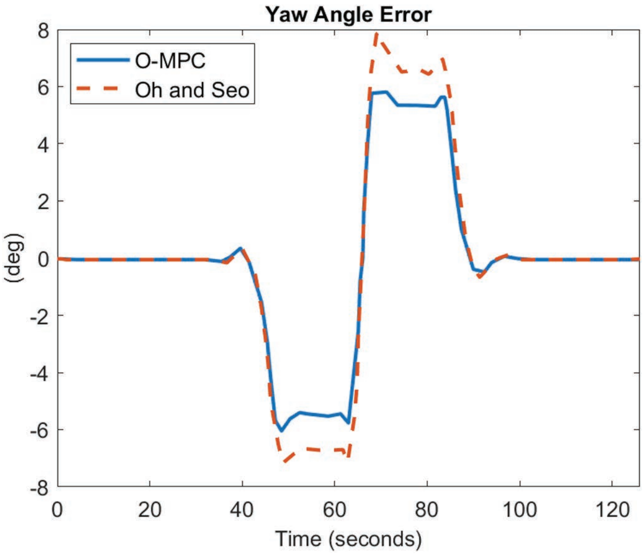

Fig 22. Yaw Angle Error at 25 km/h.

https://doi.org/10.1371/journal.pone.0324720.g022

system consistently outperformed the system by Oh and Seo [3] in terms of steering efficiency, dynamic stability, and tracking performance in all scenarios. This demonstrates the effectiveness of the O-MPC approach in enhancing the overall performance of multi-axle crane steering systems.

5. Conclusion and recommendation

This study successfully developed an Optimal Model Predictive Control (O-MPC) steering system for multi-axle cranes, incorporating a Smell Agent Optimization (SAO)-based weighting factor to enhance steering efficiency, dynamic stability, and path tracking performance. The O-MPC system was rigorously tested and compared against an existing system by

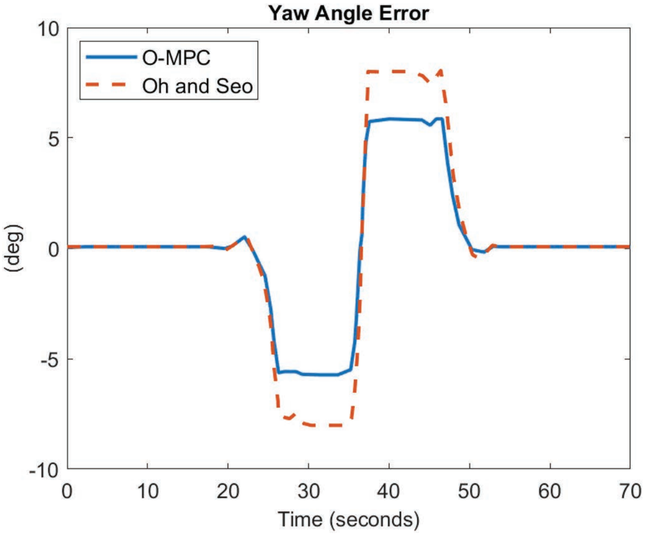

Fig 23. Yaw Angle Error at 45 km/h.

https://doi.org/10.1371/journal.pone.0324720.g023

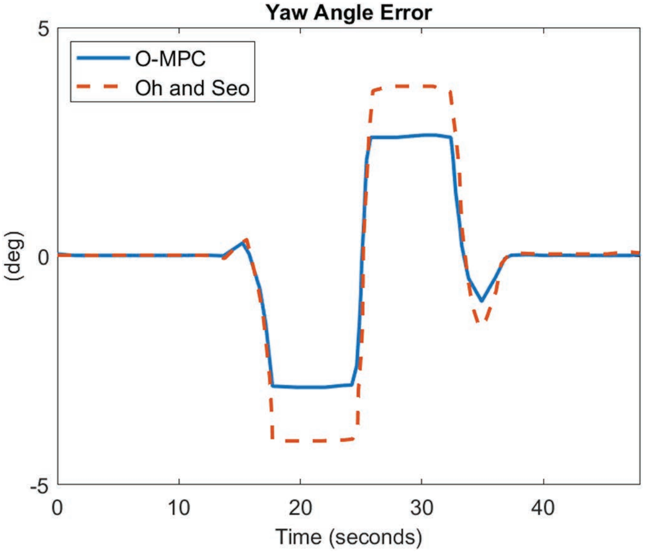

Fig 24. Yaw Angle Error at 65 km/h.

https://doi.org/10.1371/journal.pone.0324720.g024

Oh and Seo [3] across three different driving speed scenarios. Results demonstrated that the O-MPC system significantly reduced driver steering effort, improved dynamic stability, and achieved superior path tracking in all scenarios. These findings confirm the effectiveness of the proposed system, providing a comprehensive solution to the challenges of multi-axle

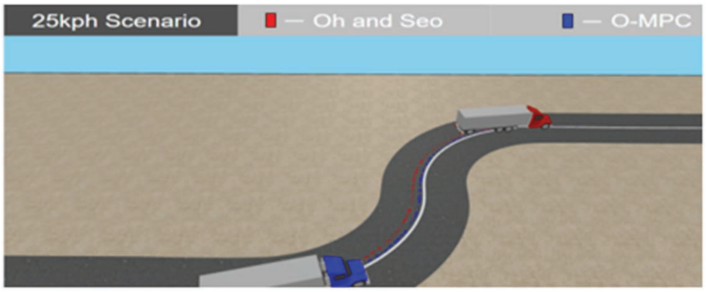

Fig 25. Tracking Result in 3D Simulation at 25 km/h.

https://doi.org/10.1371/journal.pone.0324720.g025

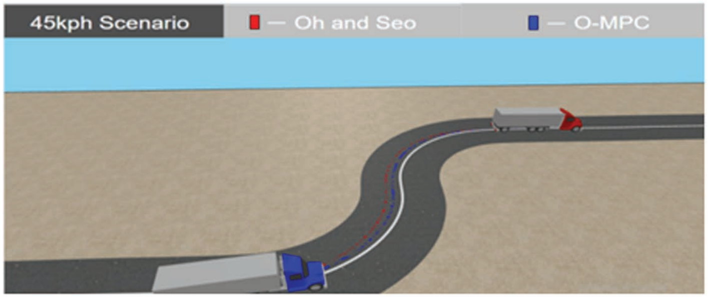

Fig 26. Tracking Result in 3D Simulation at 45 km/h.

https://doi.org/10.1371/journal.pone.0324720.g026

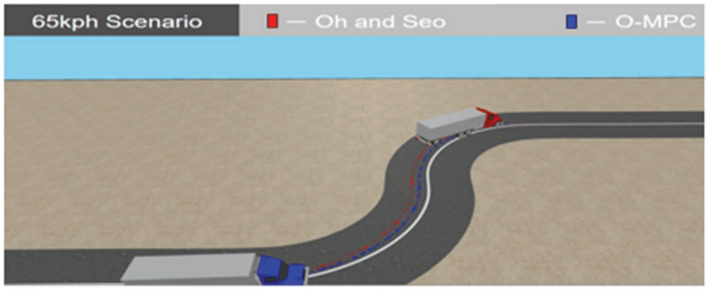

Fig 27. Tracking Result in 3D Simulation at 65 km/h.

https://doi.org/10.1371/journal.pone.0324720.g027

Table 6. Performance Comparison for 25 km/h Scenario.

Metric	O-MPC	Benchmark	Performance Improvement (%)
**Steering Efficiency (m/deg)**	361	317	13.88
**Yaw rate (deg/s)**	2.33	2.39	2.29
**Lateral Error (m)**	0.15	0.21	26.78
**Yaw angle error (deg)**	2.51	3.41	26.35

https://doi.org/10.1371/journal.pone.0324720.t006

Table 7. Performance Comparison for 45 km/h Scenario.

Metric	O-MPC	Benchmark	Performance Improvement (%)
**Steering Efficiency (m/deg)**	330	226	46.02
**Yaw rate (deg/s)**	4.18	4.22	1.03
**Lateral Error (m)**	0.23	0.32	27.52
**Yaw angle error (deg)**	2.11	2.91	27.44

https://doi.org/10.1371/journal.pone.0324720.t007

Table 8. Performance Comparison for 65 km/h Scenario.

Metric	O-MPC	Benchmark	Performance Improvement (%)
**Steering Efficiency (m/deg)**	316	267	18.35
**Yaw rate (deg/s)**	5.77	6.02	4.17
**Lateral Error (m)**	0.26	0.38	29.25
**Yaw angle error (deg)**	1.70	2.39	28.93

https://doi.org/10.1371/journal.pone.0324720.t008

crane steering control. A 3D simulation model of the multi-axle crane was also developed in AnyLogic, which provided a visual interpretation of the tracking results. This work addresses the limitations of previous approaches and contributes a robust control strategy applicable across diverse driving speed conditions, paving the way for future research and real-world implementation.

As a recommendation for further work, the developed control strategy can be deployed on several scenarios to create a broader baseline range for additional comparison with other control strategies. Furthermore, a non-linear model of the multi-axle crane can be developed to reflect changes in actuator response characteristics and vehicle dynamics. This could lead to enhanced control accuracy under extreme maneuvering conditions. However, it could also lead to increased computational cost, which can also be studied to determine the appropriate level of model complexity for implementation. Another direction of work could be to investigate the use of adaptive or self-tuning approaches within the SAO-based weighting factor to improve the performance and robustness of the controller. The prospects for using adaptive, optimized MPC could also be investigated. This could be useful in improving the robustness of the MPC controller to parameter variation, actuator saturation, and external disturbances. Finally, further studies would focus on testing the proposed O-MPC steering system's physical multi-axle crane prototypes. This will allow for the assessment of the controller's performance under real-world conditions, such as variable loads and other disturbances. It would also provide a platform for testing other works recommended in this section. Within this realm, research would be carried out to optimize the controller's computational load. This could be achieved by investigating hardware-in-the-loop (HIL) testing and dedicated embedded platforms for resource-constrained systems. Another important study is to benchmark O-MPC with other advanced controllers, such as fuzzy controller, event-triggered control and other adaptive control techniques.

Supporting information

S1. AllFigData.mat.
(MAT)

S2. Fig10data.
(CSV)

S3. Fig11data.
(CSV)

S4. Fig12data.
(CSV)

S5. Fig13OH.
(CSV)

S6. Fig14OH.
(CSV)

S7. Fig15OH.
(CSV)

S8. Fig16OH.
(CSV)

S9. Fig17OH.
(CSV)

S10. Fig18OH.
(CSV)

S11. Fig19OH.
(CSV)

S12. Fig20OH.
(CSV)

S13. Fig21OH.
(CSV)

S14. Fig22OH.
(CSV)

S15. Fig23OH.
(CSV)

S16. Fig24OH.
(CSV)

Author contributions

Conceptualization: Abdulhakeem Muhammed Ali, Yusuf Abubakar Sha'aban, Ahmed Tijani Salawudeen, Muhammed Bashir Mu'azu.

Data curation: Abdulhakeem Muhammed Ali.

Formal analysis: Abdulhakeem Muhammed Ali, Yusuf Abubakar Sha'aban, Zaharuddeen Haruna, Bilyamin Muhammad, Abdullah Alharthi.

Investigation: Ahmed Tijani Salawudeen.

Methodology: Abdulhakeem Muhammed Ali, Yusuf Abubakar Sha'aban, Ahmed Tijani Salawudeen, Zaharuddeen Haruna, Muhammed Bashir Mu'azu.

Resources: Yusuf Abubakar Sha'aban, Ahmed Tijani Salawudeen, Bilyamin Muhammad, Abdullah Alharthi.

Software: Abdulhakeem Muhammed Ali, Yusuf Abubakar Sha'aban, Ahmed Tijani Salawudeen.

Supervision: Yusuf Abubakar Sha'aban, Ahmed Tijani Salawudeen, Muhammed Bashir Mu'azu.

Validation: Zaharuddeen Haruna, Bilyamin Muhammad, Abdullah Alharthi.

Visualization: Zaharuddeen Haruna, Bilyamin Muhammad, Abdullah Alharthi.

Writing – original draft: Abdulhakeem Muhammed Ali.

Writing – review & editing: Abdulhakeem Muhammed Ali, Yusuf Abubakar Sha'aban, Ahmed Tijani Salawudeen, Zaharuddeen Haruna, Muhammed Bashir Mu'azu, Abdullah Alharthi.

References

1. Qin G, Wu J, Zhang Y, Chen L. Multidisciplinary Co-Simulation of All-Terrain Crane With the Hydro-Pneumatic Suspension and Multi-Bridges Steering System. Volume 4: 12th International Conference on Advanced Vehicle and Tire Technologies; 4th International Conference on Micro- and Nanosystems. ASMEDC; 2010. p. 111–120. https://doi.org/10.1115/detc2010-28195
2. Oh K, Seo J, Kim J-G, Yi K. MPC-based approach to optimized steering for minimum turning radius and efficient steering of multi-axle crane. Int J Control Autom Syst. 2017;15(4):1799–813. https://doi.org/10.1007/s12555-015-0472-6
3. Oh K, Seo J. Model predictive control–based approach for assist wheel control of a multi-axle crane to improve steering efficiency and dynamic stability. Proceedings of the Institution of Mechanical Engineers, Part D: Journal of Automobile Engineering. 2018;233(4):948–60. https://doi.org/10.1177/0954407018756557
4. Oh K, Seo J, Kim J, Yi K. An investigation on steering optimization for minimum turning radius of multi-axle crane based on MPC algorithm. 2015 15th International Conference on Control, Automation and Systems (ICCAS). IEEE; 2015. p. 1974–1977. https://doi.org/10.1109/iccas.2015.7364691
5. Du H, Wei J, Fang J. The design, simulation, and experiment of high-accuracy multi-axle electro-hydraulic control servo steering system. Advances in Mechanical Engineering. 2016;8(10). https://doi.org/10.1177/1687814016674383
6. Salawudeen AT, Mu'azu MB, Sha'aban YA, Adedokun AE. A Novel Smell Agent Optimization (SAO): An extensive CEC study and engineering application. Knowledge-Based Systems. 2021;232:107486. https://doi.org/10.1016/j.knosys.2021.107486
7. Salawudeen AT, Meadows OA, Yahaya B, Mu'azu MB. A novel solid waste instance creation for an optimized capacitated vehicle routing model using discrete smell agent optimization algorithm. Systems and Soft Computing. 2024;6:200099. https://doi.org/10.1016/j.sasc.2024.200099
8. Vishnoi S, Nikolovski S, Raju M, Kirar MK, Rana AS, Kumar P. Frequency Stabilization in an Interconnected Micro-Grid Using Smell Agent Optimization Algorithm-Tuned Classical Controllers Considering Electric Vehicles and Wind Turbines. Energies. 2023;16(6):2913. https://doi.org/10.3390/en16062913
9. Bhukya M, Injeti SK, Ogirala RP, Kotte S. Real-Time Simulation and Validation of Interconnected Microgrid Load Frequency Control With Uncertainties Using PDN-Iλ Dμ Controller Based on Improved Smell Agent Optimization. IEEE Trans Ind Inf. 2024;20(5):7238–48. https://doi.org/10.1109/tii.2024.3353825
10. Okoronkwo JO, Mati A, Yusuf J, Olarinoye G, Abubakar AS. Development of Fractional Order Controller for Load Frequency Control of Multi Area Interconnected Microgrid Power System. 2021 1st International Conference on Multidisciplinary Engineering and Applied Science (ICMEAS). IEEE; 2021. p. 1–5. https://doi.org/10.1109/icmeas52683.2021.9739813
11. Abba AM, Karataev T, Thomas S, Ali AM, Yau I, Mikail SA. Optimal PID Controller Tuning for DC Motor Speed Control Using Smell Agent Optimization Algorithm. FUOYEJET. 2022;7(1). https://doi.org/10.46792/fuoyejet.v7i1.740
12. Mas'ud AA, Salawudeen AT, Umar AA, Aziz AS, Shaaban YA, Muhammad-Sukki F, et al. A Quasi oppositional smell agent optimization and its levy flight variant: A PV/Wind/battery system optimization application. Applied Soft Computing. 2023;147:110813. https://doi.org/10.1016/j.asoc.2023.110813
13. Duankhan P, Sunat K, Soomlek C. An Adaptive Smell Agent Optimization with Binomial Crossover and Linnik Flight for Engineering Optimization Problems. 2024 28th International Computer Science and Engineering Conference (ICSEC). IEEE; 2024. p. 1–6. https://doi.org/10.1109/icsec62781.2024.10770710
14. Kotte S, Injeti SK, Thunuguntla VK, Kumar PP, Nuvvula RSS, Dhanamjayulu C, et al. Energy curve based enhanced smell agent optimizer for optimal multilevel threshold selection of thermographic breast image segmentation. Sci Rep. 2024;14(1):21833. https://doi.org/10.1038/s41598-024-71448-6 PMID: 39294221
15. Arumugam M, Thiyagarajan A, Adhi L, Alagar S. Crossover smell agent optimized multilayer perceptron for precise brain tumor classification on MRI images. Expert Systems with Applications. 2024;238:121453. https://doi.org/10.1016/j.eswa.2023.121453
16. Di Cairano S, Kolmanovsky IV. Automotive Applications of Model Predictive Control. Control Engineering. Springer International Publishing. 2018. p. 493–527. https://doi.org/10.1007/978-3-319-77489-3_21

17. Sha'aban YA, Lennox B, Laurí D. PID versus MPC Performance for SISO Dead-time Dominant Processes. IFAC Proceedings Volumes. 2013;46(32):241–6. https://doi.org/10.3182/20131218-3-in-2045.00054
18. Sha'aban YA. The Effect of Dead-Time and Damping Ratio on the Relative Performance of MPC and PID on Second Order Systems. Applied Sciences. 2023;13(2):1138. https://doi.org/10.3390/app13021138
19. Sha'aban YA, Tahir F, Masding PW, Mack J, Lennox B. Control Improvement Using MPC: A Case Study of pH Control for Brine Dechlorination. IEEE Access. 2018;6:13418–28. https://doi.org/10.1109/access.2018.2810813
20. Sha'aban Y, Ikpehai A, Adebisi B, Rabie K. Bi-Directional Coordination of Plug-In Electric Vehicles with Economic Model Predictive Control. Energies. 2017;10(10):1507. https://doi.org/10.3390/en10101507
21. Musa MJ, Sudin S, Mohamed Z, Sha'Aban YA, Usman AD, Hassan AU. An improved topology model for two-vehicle look-Ahead and rear-vehicle convoy control. 2017 IEEE 3rd International Conference on Electro-Technology for National Development, NIGERCON 2017. 2018. p. 548–553. https://doi.org/10.1109/nigercon.2017.8281924
22. Harbi I, Rodriguez J, Liegmann E, Makhamreh H, Heldwein ML, Novak M, et al. Model-Predictive Control of Multilevel Inverters: Challenges, Recent Advances, and Trends. IEEE Trans Power Electron. 2023;38(9):10845–68. https://doi.org/10.1109/tpel.2023.3288499
23. Joshal KS, Gupta N. Microgrids with Model Predictive Control: A Critical Review. Energies. 2023;16(13):4851. https://doi.org/10.3390/en16134851
24. Katayama S, Murooka M, Tazaki Y. Model predictive control of legged and humanoid robots: models and algorithms. Advanced Robotics. 2023;37(5):298–315. https://doi.org/10.1080/01691864.2023.2168134
25. Sha'aban YA. Automatic Tuning of MPC using Genetic Algorithm with Historic Process Data. 2022 IEEE 18th International Colloquium on Signal Processing & Applications (CSPA). IEEE; 2022. p. 329–334. https://doi.org/10.1109/cspa55076.2022.9782011
26. Sha'aban YA. Model predictive control from routine plant data. IFAC Journal of Systems and Control. 2019;8:100050. https://doi.org/10.1016/j.ifacsc.2019.100050
27. Hu X, Chen H, Gong X, Hu Y, Wang P. Embedded Model Predictive Control for Torque Distribution Optimization of Electric Vehicles. IEEE/ASME Trans Mechatron. 2025;30(1):96–107. https://doi.org/10.1109/tmech.2024.3383230
28. Guo N, Lenzo B, Zhang X, Zou Y, Zhai R, Zhang T. A Real-Time Nonlinear Model Predictive Controller for Yaw Motion Optimization of Distributed Drive Electric Vehicles. IEEE Trans Veh Technol. 2020;69(5):4935–46. https://doi.org/10.1109/tvt.2020.2980169
29. Heng D, Bin H. Recent Patents on the Steering System of Multi-Axle Vehicle. MENG. 2015;8(2):112–9. https://doi.org/10.2174/2212797608666150528213236
30. Grigoryev I. The new Big Book of Simulation Modeling. 2020 [cited 13 Sep 2024]. Available: https://www.anylogic.com/blog/the-new-big-book-of-simulation-modeling/
31. Yakimov IM, Trusfus M V, Mokshin V V, Kirpichnikov AP. AnyLogic, ExtendSim and Simulink Overview Comparison of Structural and Simulation Modelling Systems. 2018 3rd Russian-Pacific Conference on Computer Technology and Applications (RPC). IEEE; 2018. p. 1–5. https://doi.org/10.1109/rpc.2018.8482152
32. Oh K, Seo J, Han J-W. LQR-based adaptive steering control algorithm of multi-axle crane for improving driver's steering efficiency and dynamic stability. 2016 16th International Conference on Control, Automation and Systems (ICCAS). IEEE; 2016. p. 792–796. https://doi.org/10.1109/iccas.2016.7832403
33. Shen YH, Gao Y, Xu T. Multi-axle vehicle dynamics stability control algorithm with all independent drive wheel. IntJ Automot Technol. 2016;17(5):795–805. https://doi.org/10.1007/s12239-016-0078-x
34. Jagirdar V V, Maskar VP, Trikande MW. Steering strategy for a multi-axle wheeled vehicle. 2017 International Conference on Advances in Mechanical, Industrial, Automation and Management Systems (AMIAMS). IEEE; 2017. p. 164–171. https://doi.org/10.1109/AMIAMS.2017.8069206
35. Seo J-H, Oh K-S, Noh H-J. Model predictive control–based steering control algorithm for steering efficiency of a human driver in all-terrain cranes. Advances in Mechanical Engineering. 2019;11(6):168781401985978. https://doi.org/10.1177/1687814019859783
36. Milani S, Samim Ünlüsoy Y, Marzbani H, Jazar RN. Semitrailer Steering Control for Improved Articulated Vehicle Manoeuvrability and Stability. Nonlinear Engineering. 2019;8(1):568–81. https://doi.org/10.1515/nleng-2018-0124
37. Seo J, Cha M, Oh K, Park Y-J, Kwon TJ. Development of Steering Control Algorithms with Self-tuning Fuzzy PID for All-terrain Cranes. 2020 20th International Conference on Control, Automation and Systems (ICCAS). IEEE; 2020. p. 727–730. https://doi.org/10.23919/iccas50221.2020.9268346
38. Yazıcı CB, Kutluay E, Ünlüsoy YS. Steering optimization for multiaxle vehicles with multiaxle steering. J Mech Sci Technol. 2021;35(8):3303–11. https://doi.org/10.1007/s12206-021-0704-z
39. Norouzi A, Asl HA, Kazemi R, Hafshejani PF. Adaptive sliding mode control of a four-wheel-steering autonomous vehicle with uncertainty using parallel orientation and position control. IJHVS. 2020;27(4):499. https://doi.org/10.1504/ijhvs.2020.109290
40. Nguyen A-T, Sentouh C, Zhang H, Popieul J-C. Fuzzy Static Output Feedback Control for Path Following of Autonomous Vehicles With Transient Performance Improvements. IEEE Trans Intell Transport Syst. 2020;21(7):3069–79. https://doi.org/10.1109/tits.2019.2924705
41. Aslam MS, Bilal H, Chang W, Yahya A, Badruddin IA, Kamangar S, et al. Indirect adaptive observer control (I-AOC) design for truck–trailer model based on T–S fuzzy system with unknown nonlinear function. Complex Intell Syst. 2024;10(5):7311–31. https://doi.org/10.1007/s40747-024-01544-7

42. Ding F, Zhu K, Liu J, Peng C, Wang Y, Lu J. Adaptive Memory Event-Triggered Output Feedback Finite-Time Lane-Keeping Control for Autonomous Heavy Truck With Roll Prevention. IEEE Trans Fuzzy Syst. 2024;32(12):6607–21. https://doi.org/10.1109/tfuzz.2024.3454344
43. Xu Y, Guo G, Yu S. Resilient observer-based sliding mode control of connected vehicles with denial-of-service attacks. Journal of the Franklin Institute. 2022;359(7):2886–905. https://doi.org/10.1016/j.jfranklin.2022.02.036
44. Mohammed A, Ramlal C, Marine L, Muddeen F. Resilient Event Triggered Interval Type-2 Fuzzy Sliding Mode Control for Connected and Autonomous Vehicles Subjected to Multiple Cyber Attacks. Journal of Advanced Transportation. 2024;2024(1). https://doi.org/10.1155/2024/2790548
45. Qin SJ, Badgwell TA. A survey of industrial model predictive control technology. Control Engineering Practice. 2003;11(7):733–64. https://doi.org/10.1016/s0967-0661(02)00186-7
46. Wang L. A Tutorial on Model Predictive Control: Using a Linear Velocity-Form Model. Dev Chem Eng Mineral Process. 2004;12(5–6):573–614. https://doi.org/10.1002/apj.5500120511
47. Rossiter JA. Model-Based Predictive Control. Rossiter JA, editor. CRC Press; 2017. https://doi.org/10.1201/9781315272610
